# Exposition aux poussières de manioc, maïs, soja, et santé respiratoire des meuniers artisanaux de Lubumbashi (République démocratique du Congo)

**DOI:** 10.48327/mtsi.v4i4.2024.610

**Published:** 2024-12-11

**Authors:** Léon Kabamba NGOMBE, Nlandu Roger NGATU, Kazadi Sha NGOMBE, Stanislas Wembonyama OKITOTSHO, Michel Nzaji KABAMBA, Jean-Baptiste Kakoma SAKATOLO, Oscar Luboya NUMBI

**Affiliations:** 1 Université de Kamina, École de santé publique, Département de la recherche, 273, Kamina, République démocratique du Congo; 2 Congo-Japan NCDs Research Team, Kongo central province, République démocratique du Congo; 3 Kagawa University Faculty of Medecine, Kagawa 761-0793, Japon; 4 Institut supérieur des techniques médicales de Lubumbashi (ISTM-Lubumbashi), République démocratique du Congo; 5 Université de Lubumbashi, École de santé publique de Lubumbashi, République démocratique du Congo

**Keywords:** Exposition aux poussières, Meuniers, Santé respiratoire, Spirométrie, Lubumbashi, République démocratique du Congo, Afrique subsaharienne, Dust exposure, Millers, Respiratory health, Spirometry, Lubumbashi, Democratic Republic of Congo, Sub-Saharan Africa

## Abstract

**Objectifs:**

Ce travail avait pour objectif de déterminer la prévalence des symptômes respiratoires, leurs déterminants, ainsi que l’état de la fonction respiratoire des meuniers exposés aux poussières de manioc, maïs et soja à Lubumbashi, en République démocratique du Congo (RDC), en comparaison avec un groupe des travailleurs non-exposés.

**Méthodes:**

Une étude transversale descriptive et analytique a été menée en 2015 à laquelle ont pris part 288 meuniers et 118 agents (n = 406) d'une agence de sécurité (groupe contrôle) à Lubumbashi, en RDC. Les participants ont été examinés sur leur lieu de travail. Les informations obtenues concernant la santé respiratoire ont été recueillies sur la base d'un questionnaire standardisé. Une spirométrie a été réalisée chez chaque participant.

**Résultats:**

L’âge moyen était de 27,6 ± 9 années chez les meuniers et 28,5 ± 7 chez les témoins. La durée journalière de travail était de 12,1±1,7 heures pour les meuniers contre 14,4±6,2 heures chez les témoins. Aucune différence significative n'a été trouvée en comparant les deux groupes. Cependant la prévalence des symptômes respiratoires était plus élevée chez les meuniers que chez le groupe contrôle, notamment pour les sifflements (1,9 fois plus), la sensation de gêne respiratoire (2,1 fois), l'essoufflement au repos (6 fois plus), l'essoufflement à l'effort (6,4 fois plus), la bronchite chronique (6,2 fois plus), la toux (5,3 fois plus) et la présence d'expectorations le matin (5,1 fois plus). Une association a donc été trouvée entre la profession meunière et tous les symptômes respiratoires. Les données spirométriques ont montré que le volume expiratoire maximal à la seconde (VEMS), le débit expiratoire de pointe (DEP) (p<0,05) et le rapport de Tiffeneau (VEMS/CVF) (p<0,001) étaient significativement réduits chez les meuniers comparés aux témoins.

**Conclusion:**

Cette étude a montré une prévalence élevée des symptômes respiratoires avec atteinte de la fonction pulmonaire chez les meuniers de Lubumbashi et suggère la nécessité de mettre en place des mesures préventives devant réduire l'exposition dans les meuneries.

## Abstract (en)

**Objectives:**

The aim of this study was to determine the prevalence of respiratory symptoms, their determinants, and the state of respiratory function in millers exposed to cassava, maize, and soybean dust in Lubumbashi, Democratic Republic of Congo (DRC), compared with a group of unexposed workers.

**Methods:**

A descriptive and analytical cross-sectional study was conducted in 2015 on 288 millers and 118 agents (n = 406) from a security agency (control group) in Lubumbashi, DRC. Participants were examined at their place of work. Respiratory health information was collected using a standardized questionnaire. Spirometry was performed on each participant.

**Results:**

Mean age was 27.6 ± 9 years in millers and 28.5 ± 7 years in controls. Daily working hours were 12.1±1.7 hours for millers and 14.4±6.2 hours for controls. No significant differences were found when comparing the two groups. However, the prevalence of respiratory symptoms was higher in the millers than in the controls, especially wheezing (1.9 times higher), dyspnea (2.1 times higher), breathlessness at rest (6 times higher), breathlessness on exertion (6.4 times higher), chronic bronchitis (6.2 times higher), cough (5.3 times higher) and morning sputum (5.1 times higher). Thus, an association was found between the milling occupation and all respiratory symptoms. Spirometric data showed that forced expiratory volume in one second (FEV1), peak expiratory flow (PEF) (p<0.05) and Tiffeneau ratio (FEV1/FVC) (p<0.001) were significantly reduced in millers compared to controls.

**Conclusions:**

This study showed a high prevalence of respiratory symptoms with impaired lung function among millers in Lubumbashi, suggesting the need for preventive measures to reduce exposure in mills.

## Introduction

Les effets respiratoires des poussières des farines et d'autres variétés de poussières chez les sujets exposés, du fait de leur travail, dans les petites et grandes industries de meunerie sont connus de longue date [3, 35]. Les maladies respiratoires provoquées par les poussières environnementales sont influencées par le type des poussières et la durée d'exposition [22]. Plusieurs rapports scientifiques suggèrent que le manque de moyens de protection contre les poussières en milieu agricole peut conduire à la fibrose pulmonaire [12, 32, 33]. Les poussières produites par les manipulations des grains de céréales peuvent contenir les produits suivants : des fragments de céréales (friction entre les grains, résidus de broyage, enveloppes des grains, etc.), des pollens, des bactéries (endotoxines), des champignons (mycélium, spores, toxines), des insectes et invertébrés (insectes des céréales, mites), des débris provenant de mammifères et d'oiseaux (poils, plumes, excréments), des poussières minérales (silice, etc.), des pesticides [13]. Toutes ces poussières varient suivant le type de grains, les conditions d'entreposage, les conditions de température et d'humidité.

**Figure 1:**
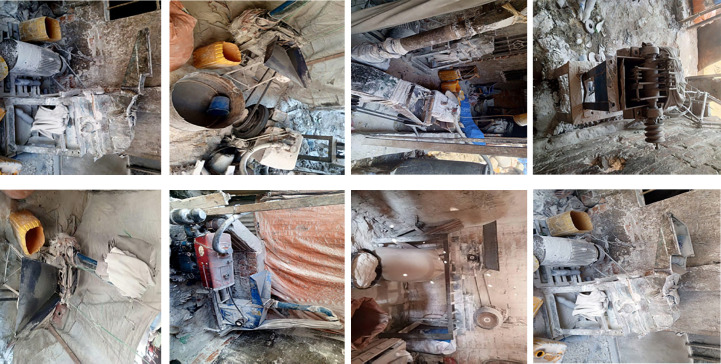
Matériels vétustes dans une meunerie artisanale (à suivre)

**Figure 2:**
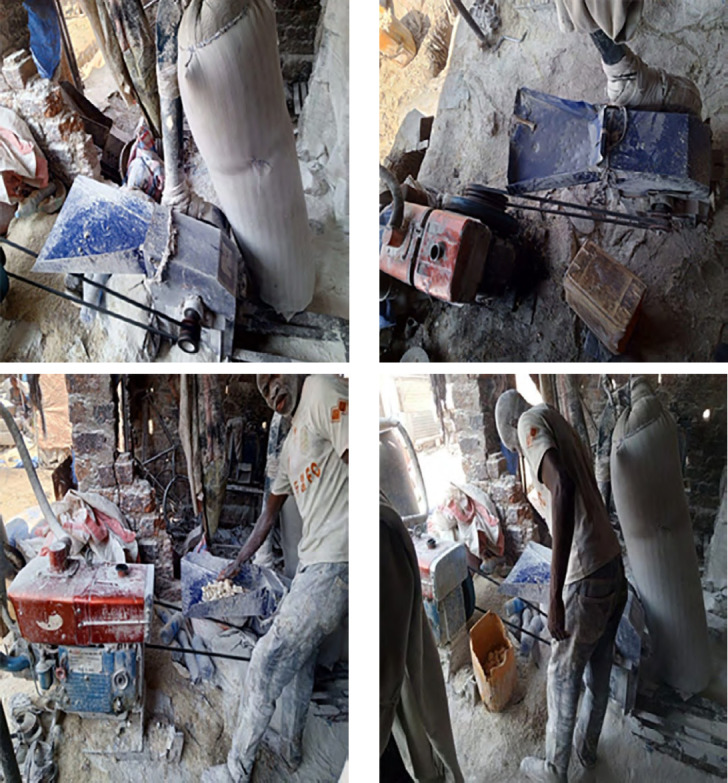
Moulins à manioc

D'autre part, les grandes quantités de poussières retrouvées dans l'air ambiant dans les meuneries artisanales sont dues entre autres à la vétusté des matériels, aux manipulations des céréales, particulièrement lors du transport, au déversement des grains dans les machines, pendant le processus de leur broyage et de l'ensachage des farines (Fig. 1, 2, 3 et 4). Cependant, il est connu que l'exposition aux poussières dans l'environnement professionnel peut être réduite ou éliminée par l'amélioration des matériels de transformation, les systèmes de ventilation, les bonnes conditions du travail et l'usage des matériels de protection individuelle [10]. L'exposition aux poussières organiques et aux endotoxines causent différentes pathologies respiratoires (asthme, alvéolite, bronchites chroniques, pneumopathies toxiques) [11, 14, 19, 25], ainsi que des défaillances aiguës ou chroniques de la fonction respiratoire [31, 34, 37, 38].

Commentaires : Présentation des équipements vétustes et du milieu de travail des meuniers artisanaux congolais qui ne répondent pas aux exigences de la santé au travail. On observe aussi beaucoup des poussières des céréales partout.

**Figure 3:**
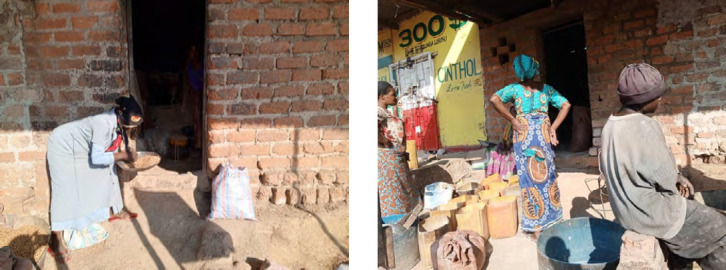
Vue de face de l'entrée d'une meunerie artisanale

Commentaires : Pas de fenêtres d aération, les céréales sont mis dans des bidons coupés.

**Figure 4:**
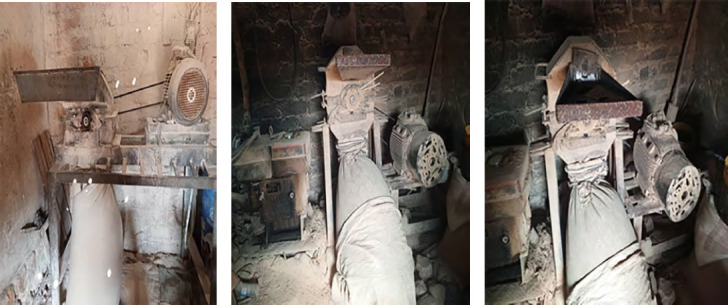
Moulins pour soja

Commentaires : Moulin à soja. Matériel vétuste, beaucoup des poussières de soja dans la pièce qui n'est pas aérée.

Les farines de manioc et de maïs figurent parmi les aliments de base tant en R**é**publique d**é**mocratique du Congo (RDC) que dans les autres pays de la r**é**gion de l’Afrique centrale. Ainsi, la meunerie est devenue en RDC une activité économique répandue et lucrative. Toutefois, elle n'est pas réglementée et comporte un certain nombre de risques de santé. Elle se pratique généralement dans un espace clos, non aéré et sans usage de moyens de protection contre les poussières, les vibrations du moulin ou les gaz d’échappement des moteurs thermiques.

La littérature révèle les symptômes et troubles respiratoires chez les meuniers, mais, à notre connaissance, aucune étude scientifique explorant leur état respiratoire n'a été menée ou publiée en RDC ou en Afrique centrale. Le but de ce travail était de déterminer la prévalence des symptômes respiratoires et leurs déterminants chez les meuniers artisanaux exposés aux poussières de manioc, maïs et soja dans la ville de Lubumbashi, province du Katanga, en RDC.

## Matériels et méthodes

### Type d’étude, cadre et échantillon d’étude

Une étude transversale à visée analytique a été menée en 2015 sur une période de trois mois, allant du 1^er^ mai au 31 juillet 2015 dans les sept communes de la ville de Lubumbashi, province du Katanga.

La population d’étude était des meuniers artisanaux (groupe des exposés) et des agents d'une société locale de gardiennage (groupe témoin) travaillant dans les mêmes quartiers que les meuniers artisanaux, dans la ville de Lubumbashi [28]. En l'absence de service de médecine du travail, le recrutement des enquêtés a été fait sur leurs lieux de travail respectifs. Un total de 510 meuneries artisanales a été identifié dans les 7 communes de la ville de Lubumbashi (chaque commune a été considérée comme un site) grâce à l'appui des chefs des quartiers et des représentants des meuniers. Seules 390 meuneries artisanales étaient opérationnelles pendant la période de notre étude. La taille de l’échantillon était fonction du nombre de meuniers que nous avons trouvé sur le lieu de service, tous les jours impairs de la semaine, et recensés à partir des listes élaborées par les chefs de quartiers ou les représentants des meuniers artisanaux. Nous avons procédé à un échantillonnage exhaustif de tous les meuniers de la ville de Lubumbashi. Au total, 350 meuniers artisanaux dont l'ancienneté dans le métier était supérieure à 6 mois ont été recrutés dans cette étude (dont 2 femmes); parmi eux, 288 remplissant les critères d'inclusion (présence sur le lieu de travail le jour du recrutement, consentement éclairé et participation volontaire) ont été sélectionnés pour prendre part à l'enquête. Chaque meunier a reçu une fiche d'enquête anonyme qui a également servi à consigner les données de l'examen clinique.

Le groupe témoin comportait 125 agents de sécurité recrutés de manière exhaustive, avec plus de six mois d'ancienneté et un niveau socioéconomique similaire à celui des meuniers artisanaux. Sept témoins n'ont pas accepté de prendre part à cette étude et en ont été exclus.

### Questionnaire d'enquête

L’étude a consisté en une analyse des conditions de travail (questionnaire, examen physique, et spirométrie). Les travailleurs ont été examinés sur leur lieu de travail respectif. Les informations obtenues sur la santé des meuniers et des témoins ont été recueillies sur base d'un questionnaire standardisé comprenant 7 rubriques de questionnaires, notamment : un questionnaire général portant sur les caractéristiques sociodémographiques et professionnelles, un questionnaire sur les symptômes respiratoires [5], un questionnaire sur la rhinite allergique [2], un questionnaire standardisé sur les troubles du sommeil et de la vigilance associé à l’échelle de somnolence d’Epworth [15], le questionnaire de Berlin [27], un questionnaire neurologique [21]. Seul le questionnaire sur les symptômes respiratoire a été utilisé dans notre enquête [2, 5].

### Définitions des pathologies ciblées et exploration respiratoire fonctionnelle

La bronchite chronique a été définie selon les critères de l’Organisation mondiale de la santé (OMS) comme une toux et des expectorations chroniques survenant au moins trois mois par an depuis au moins deux ans. Les rhinites ont été classiquement caractérisées sur la base d'un score quantitatif SFAR *(Score For Allergic Rhinitis).* Le poids a été pris au moyen d'une balance calibrée et vérifiée; la taille a été mesurée avec une toise. L'enregistrement de la pression artérielle au moyen d'un appareil électronique (OMRON Hem8402) a été effectué au bras droit soutenu à hauteur du cœur, le sujet s’étant reposé pendant au moins 10 minutes en position assise.

La spirométrie a été réalisée chez les participants ayant librement consenti de passer ce test, dont 160 meuniers et 90 témoins. Elle a été effectuée à l'extérieur de l'atelier à l'aide d'un appareil portable « *Easy One »* ne nécessitant pas de calibration. Chaque sujet a fait au moins trois essais successifs, et le meilleur résultat a été retenu en tenant comptant des exigences du protocole de l’ATS/ERS 2005. Les paramètres ci-dessous ont été mesurés : la capacité vitale forcée (CVF), le volume expiratoire maximum en une seconde (VEMS), le rapport de Tiffeneau (VEMS/CVF), le débit expiratoire de pointe (DEP), le débit expiratoire médian (DEM), le débit expiratoire maximum à 25, 50 et 75 % de la capacité vitale (DEM25, DEM50, DEM75).

### Considérations éthiques

La confidentialité et l'anonymat ont été garantis aux personnes qui ont répondu au questionnaire. Les enquêteurs ont bénéficié d'une formation au préalable et l'enquête a été réalisée par entretien direct entre les enquêteurs et les personnes incluses dans l’étude. Le consentement éclairé des sujets a été obtenu.

### Analyses statistiques des données de l’étude

Le logiciel EPI info7 a permis l'encodage des données. Toutes les analyses ont été effectuées à l'aide du logiciel Stata (Stata Corporation Inc., Texas, USA). L’étude statistique a reposé sur le test « T » de Student pour la comparaison des moyennes en rapport avec les paramètres de la fonction respiratoire (spirométrie) et le test exact de Fisher pour les proportions relatives aux caractéristiques anthropométriques et socio-démographiques. Pour les variables qualitatives telles que les symptômes respiratoires, une tabulation croisée a été effectuée avec estimation des valeurs du Chi carré et du risque relatif.

D'autre part, pour déterminer l'association entre les caract**é**ristiques des meuniers et les symptômes respiratoires rapportés, une analyse multivariée avec le test de régression logistique a été privilégiée. Ce modèle a estimé la force d'association par un rapport de prévalence (Odds ratio, OR) et son intervalle de confiance à 95 % (I.C.95 %). Le seuil de signification choisi correspond à une valeur de p<0,05.

## Résultats

### Description brève des conditions de travail des meuniers

Dans les meuneries, les meuniers font face à plusieurs types d'exposition pouvant avoir des répercussions sur leur santé : les poussières de farines de manioc et de céréales (maïs, soja), les vibrations et les bruits émis par les moteurs des moulins qui sont électriques (en majorité) et vétustes, ainsi que la chaleur et les mauvaises conditions d'aération dues au manque ou à l'insuffisance du nombre des fenêtres. Les meuniers travaillent sur différents postes (maïs-manioc-soja, maïs-manioc, maïs, manioc-soja, manioc seul), et généralement dans des milieux presque fermés (sans système de ventilation adéquat). Dans certaines minoteries utilisant un moteur comme source d’énergie, le gaz d’échappement est rejeté à l'extérieur. Il est important de savoir que tous les moulins sont fixes dans des espaces clos et ne sont pas mobilisables sous des auvents. Ces minoteries artisanales n'ont jamais respecté les trois étapes de la transformation des céréales : nettoyage, mouillage et mouture. Cette enquête montre que 100 % des meuniers artisanaux n'ont pas de matériels de protection individuelle pendant le travail.

La taille de l’échantillon d’étude était de 406 participants dont 288 meuniers et 118 témoins dont les caractéristiques sont présentées dans le Tableau I.

**Tableau I:** Caractéristiques des participants

Caractéristiques des participants	Meuniers (N=288)	Témoins (N=188)
Paramètres anthropométriques, cliniques et liés au travail		
	Moyenne +/- Écart type	Moyenne +/- Écart type
âge	27,6±9	28,5±7
ancienneté (ans)	4,8±5,5	4,9±4,4#
durée de travail (heures)	12,1±1,7	14,4±6,2#
IMC	22,1±2,4	23,3±3,6#
PAS/SBP	140,3±15,9#	126,1± 17,4
PAD	90±12,1#	73,9±12,2
Paramètres socio-démographiques		
	n (%)	n (%)
état-civil		
marié	128 (44,4)	88 (74,6)*
célibataire	158 (54,9)	28 (23,7)
divorcé	2 (0,7)	0
veuf	0	2 (1,7)
éducation		
primaire	3 (1,0)	5 (4,2)
secondaire (non achevé)	244 (84,7)*	78 (66,1)
secondaire (achevé)	41 (14,2)	35 (29,7)
alcool use		
oui	125 (43,4)*	17 (14,4)
non	163 (56,6)	101 (85,6)
tabagisme		
oui	90 (31,5)*	17 (14,8)
non	196 (68,5)	98 (85,2)

IMC : Indice de masse corporelle; PAS : Pression artérielle systolique; PAD : pression artérielle diastolique)

Notes : #, valeur de P inférieure à 0,01 pour le test « T » de Student; *, valeur de P inférieur à 0,001 pour le test exact de Fisher.

BMI: Body Mass Index; SBP: Systolic Blood Pressure; DBP: Diastolic Blood Pressure)

Notes: #, P value less than 0.01 for Student's ‘T’ test; *, P value less than 0.001 for Fisher's exact test

Le Tableau II montre la prévalence des symptômes respiratoires chez les meuniers et le groupe contrôle.

**Tableau II:** Prévalence des symptômes respiratoires, avec le risque correspondant, en fonction de la profession

Symptômes respiratoires	Meuniers (N=288)	Témoins (N=188)	RR IC [95 %]	x^2^	P
Sifflements	124 (43,4)	27 (23,5)	1,88 [1,31-2,68]	14,58	< 0,001
Gêne respiratoire	100 (34,7)	20 (16,9)	2,05 [1,33-3,14]	12,70	< 0,001
Essoufflement au repos	88 (30,6)	6 (5,1)	6,01 [2,70- 13,35]	30,52	< 0,0001
Essoufflement à l'effort	94 (32,6)	6 (5,1)	6,41 [2,89-14,24]	34,23	< 0,0001
Toux	121 (42,0)	23 (19,5)	5,25 [2,35-11,73]	24,13	< 0,0001
Crachats le matin	99 (34,4)	8 (6,8)	5,07 [2,54-10,08]	32,84	< 0,0001
Bronchite chronique	76 (26,4)	5 (4,2)	6,22 [2,58-15,00]	25,72	< 0,0001
Asthme	136(47,2)	3(2,5)	18,5 [6,03-57,14]	74,22	< 0,0001
Rhinite	87 (30,2)	12 (10,2	3,82 [2,01-7,23]	18,23	< 0,0001

RR : Risque relatif; IC : Intervalle de confiance

RR: Relative risk; CI: Confidence interval

Le Tableau III montre la prévalence de chaque symptôme respiratoire tel que rapporté par les meuniers en fonction du poste de travail et des céréales auxquelles ils sont exposés.

**Tableau III:** Distribution des symptômes respiratoires chez les meuniers artisanaux en fonction du poste de travail

Symptômes respiratoires	Maïs-manioc-soja	Maïs-manioc	Maïs	Manioc-soja	Manioc	P
Sifflements						
oui	25 (55,6)	72 (39,8)	17 (38,6)	1 (50)	9 (56,3)	<0,01
non	20 (44,4)	109 (60,2)	27 (61,4)	1 (50)	7 (43,8)	
Gêne respiratoire						
oui	22 (48,9)	53 (29,3)	15 (34,1)	1 (50)	9 (56,3)	<0,01
non	23 (51,1)	128 (70,7)	29 (65,9)	1 (50)	7 (43,8)	
Essoufflement au repos						
oui	10 (22,2)	56 (30,9)	16 (36,4)	1 (50)	5 (31,3)	<0,01
non	35 (77,8)	125 (69,1)	28 (63,6)	1 (50)	11 (68,8)	
Essoufflement à l'effort						
oui	17 (37,8)	58 (32)	12 (27,3)	0 (0)	7 (43,8)	<0,01
non	28 (62,2)	123 (68)	32 (72,7)	2 (100)	9 (56,3)	
Toux						
oui	22 (48,9)	78 (43,1)	16 (36,4)	0 (0)	5 (31,3)	<0,01
non	23 (51,1)	103 (56,9)	28 (63,6)	2 (100)	11 (68,8)	
Crachats le matin						
oui	14 (31,1)	63 (34,8)	17 (38,6)	1 (50)	4 (25)	<0,01
non	31 (68,9)	118 (65,2)	27 (61,4)	1 (50)	12 (75)	
Crachats >3 mois						
oui	8 (17,8)	47 (26)	13 (29,5)	0 (0)	8 (50)	<0,01
non	37 (82,2)	134 (74)	31 (70,5)	2 (100)	8 (50)	
Asthme						
oui	19 (42,2)	87 (48,1)	23 (52,3)	0 (0)	7 (43,8)	<0,01
non	26 (57,8)	94 (51,9)	21 (47,7)	2 (100)	9 (56,3)	

Le Tableau IV montre l'association entre la profession de meunier et les manifestations respiratoires après ajustement pour les facteurs ayant une influence sur la fonction respiratoire : le tabagisme, la durée d'exposition (ancienneté dans la profession), l’âge, le niveau d’éducation et l'indice de masse corporelle.

**Tableau IV:** Prévalence des symptômes respiratoires, avec le risque correspondant, en fonction de la profession (à suivre)

Variables	Sifflements		Gêne respiratoire		Essoufflement au repos		Essoufflement à l'effort		Toux	
	OR[IC95%]	P*	OR[IC95%]	P*	OR[IC95%]	P*	OR[IC95%]	P*	OR[IC95%]	P*
Âge										
≤ 30	0,99[0,94-1,03]	0,70	0,99[0,95-1,04]	0,95	1,01[0,94-1,05]	0,98	2,75[1,23-6,14]	0,013	1,02[0,97-1,08]	0,37
> 30	1		1		1		1		1	
Ancienneté (ans)										
≤ 5	1,25[0,76-2,03]	0,369	1,13[0,67-1,9]	0,64	1,07[0,59-1,94]	0,81	1,41[1,56-1,84]	0,043	0,59[0,30-1,14]	0,12
> 5	1		1		1		1		1	
Durée de travail (heures)										
≤ 12	0,93[0,85-1,03]	0,27	0,85[0,85-1,06]	0,43	1,06[0,84-1,17]	0,17	1,22[1,44-3,32]	0,69	1,66[0,74-4,43]	0,31
> 12	1		1		1		1		1	
Tabagisme										
oui	0,96[0,59-1,55]	0,87	1,23[0,73-2,05]	0,43	0,83[0,48-1,42]	0,51	1,30[1,22-2,27]	0,35	1,03[0,58-1,82]	0,90
non	1		1		1		1		1	
Profession										
meunier	2,47[1,48-4,11]	<0,01	2,75[1,56-4,83]	<0,01	7,85[3,27-18,84]	<0,01	3,69[9,57-24,81]	<0,01	7,16[2,96-17,29]	<0,01
témoin	1		1		1		1		1	
Éducation										
primaire, secondaire (non achevé)	0,87[0,52-1,46]	0,12	1,12[0,65-1,91]	0,67	0,71[0,37-1,38]	0,32	0,61[0,31-1,18]	0,14	1,13 [0,59-2,15]	0,7
secondaire achevé, plus	1		1		1		1		1	

Variables	Crachats le matin		Bronchite chronique		Asthme		Rhinite	
	OR[IC95%]	P*	OR[IC95%]	P*	OR[IC95%]	P*	OR[IC95%]	P*
Âge								
≤ 30	1,02[0,97-1,08]	0,37	1,38[0,61-3,18]	0,40	1,03 [0,65-1,05]	0,88	0,88[0,46-1,71]	0,08
> 30	1		1		1		1	
Ancienneté (ans)								
≤ 5	1,44[1,23-1,83]	<0,01	0,66[0,34-1,28]	0,224	1,07[0,60-1,89]	0,81	1,24[0,81-1,88]	0,83
> 5	1		1		1		1	
Durée de travail (heures)								
≤ 12	1,06[0,95-1,19]	0,27	1,10[0,96-1,25]	0,14	1,74[1,10-2,29]	<0,01	1,35[0,83-2,21]	0,49
> 12	1		1		1		1	
Tabagisme								
oui	1,38[1,80-2,38]	0,245	1,55 [1,85-2,82]	0,15	1,11[0,66-1,85]	0,69	0,99[0,67-1,47]	0,23
noN	1		1		1		1	
Profession								
meunier	7,35[3,37-16,3]	<0,01	8,21[3,16-21,2]	<0,01	36,28[11,1-118,6]	<0,01	2,97[1,69-5,22]	0,001
témoin	1		1		1		1	
Éducation
primaire, secondaire (non achevé)	0,54[0,28-1,05]	0,07	1,38[1,17-1,91]	0,017	1,27[0,69-2,33]	0,44	0,61[0,31-1,18]	0,144
secondaire achevé, plus	1		1		1		1	

Le Tableau V présente la tendance des valeurs des différents paramètres de la fonction respiratoire chez les travailleurs de la meunerie et ceux du groupe contrôle. Aucun des meuniers ayant participé à l'enquête ne disposait de matériels de protection individuelle pendant le travail.

**Tableau V:** Comparaison des paramètres spirométriques entre les meuniers et les contrôles

Paramètres spirométriques	Meuniers (moyenne±écart type)	Témoins (moyenne±écart type)	T	P (T-test)
CVF (l)	4,02±0,72	4,25±0,14	0,36	0,359
VEMS (l)	3,29±0,59	3,35±0,71	2,71	0,039
VEMS/CVF (% prévu)	82,3 5±8,31	85,81±8,12	4,95	< 0,001
DEP (l/s)	8,12±1,97	8,63±1,62	1,31	0,095
VEMS/CVF (% prévu)	93,24±21,91	101,82±17,96	2,01	0,023

CVF : Capacité vitale forcée; VEMS: Volume expiratoire maximal seconde; DEP : Débit expiratoire de pointe

FVC : Forced vital capacity; FEV1: Forced expiratory volume; in 1 second PEF : Peak expiratory flow

## Discussion

Cette étude a montré que la prévalence des symptômes et des pathologies respiratoires était remarquablement élevée chez les meuniers artisanaux exposés aux poussières de manioc, maïs et soja par rapport au groupe contrôle dans la ville de Lubumbashi. Il est établi que les travailleurs exposés aux poussières peuvent également inhaler des spores ou fragments de mycélium, par exemple [8, 30], ce qui augmente le risque de développer des manifestations respiratoires [4, 6, 7, 20, 36]. L’étude révèle que les meuniers artisanaux n'avaient pas les équipements de protections requis (lunettes, masques, gants), souvent par faute de moyens, manque d’éducation ou d'information, ou du fait de la non disponibilité de ces derniers. Tous ces facteurs contribuent à la prévalence élevée des symptômes ainsi que des pathologies respiratoires chez les meuniers artisanaux congolais. Nos observations sont soutenues par la littérature [11, 22, 25] qui souligne le manque d’éducation, un niveau socio-économique bas et la non disponibilité des équipements de protection dans le secteur informel, notamment chez les ouvriers des carrières, y compris ceux du secteur agricole. Par ailleurs, nos résultats sont comparables à ceux d’études antérieures menées en RDC chez différents groupes de travailleurs exposés aux poussières [1, 10, 26, 29]. Cependant, dans notre étude, la prévalence de l'asthme dépasse de loin celle rapportée par certains auteurs [9, 24], ce qui suggère que l'asthme est une affection fréquente chez les travailleurs exposés aux poussières de céréales.

Une association a été trouvée entre la profession des meuniers artisanaux et les symptômes respiratoires qu'avaient présentés ces derniers. Selon la littérature, les composés organiques volatiles (COV) jouent un rôle dans la détérioration des voies respiratoires, tandis que les fortes concentrations des particules fines sont responsables d'altérations de l’état de santé des sujets exposés aux poussières environnementales [2, 31, 34].

L'environnement malsain (avec la présence de grandes quantités de poussières dans les locaux et même sur les machines), les conditions médiocres et non hygiéniques (exigüité des locaux, manque de ventilation, encombrement, etc.), l'exposition aux poussières de céréales pourraient expliquer aussi cette association [29]. Ce risque pourrait être majoré par les gaz d’échappement lorsqu'ils ne sont pas correctement évacués vers l'extérieur, ce que nous n'avons pas pu prendre en compte dans nos analyses. De même, le tabagisme peut influencer l'incidence, la sévérité et l’évolution spontanée de nombreuses maladies respiratoires [9, 16, 17, 24]. Dans notre étude, une prévalence élevée de consommation de tabac et d'alcool chez les meuniers artisanaux par rapport au groupe contrôle a été notée. Ce phénomène est fréquent chez les travailleurs de force exerçant leurs activités dans un contexte de conditions de travail précaires. Ce constat a également été fait par certains auteurs [29]. Le test de régression logistique suggère un impact négatif de l'exposition au tabac et aux poussières sur la santé respiratoire des meuniers artisanaux.

La réduction significative de la fonction respiratoire, notamment en ce qui concerne les paramètres tels que le VEMS et le DEP chez les meuniers artisanaux par rapport au groupe contrôle traduirait l'hypertrophie des cellules muqueuses due à l'irritation par des poussières, avec comme conséquences une augmentation de la sécrétion du mucus, la formation du bouchon muqueux et l'obstruction à l'air expiré [18, 23, 29]. Les poussières organiques diffèrent selon la nature et la composition chimique de chaque produit, ce qui peut influer sur la fréquence des manifestations respiratoires chez les travailleurs exposés. Notre étude suggère que l'exposition aux poussières de manioc et de maïs serait plus toxique pour l'appareil respiratoire qu’à celles du soja. Des études plus approfondies sont nécessaires pour éventuellement en déterminer le mécanisme. Ces travaux sont essentiels, car ils fournissent aux décideurs des politiques sanitaires et des chercheurs des informations pertinentes sur la prédominance des manifestations respiratoires, de l'environnement de travail et de l'absence des mesures préventives parmi les travailleurs de la meunerie artisanale congolaise.

Il est important de réduire les concentrations des poussières des céréales dans l'environnement de travail des meuniers, d'intégrer des systèmes de ventilation et d'aspiration des poussières dans les ateliers, d'améliorer les mesures d'hygiène, de définir nos valeurs limites d'exposition aux poussières ainsi que les dimensions des ateliers de meuniers artisanaux en RDC.

Il est aussi primordial d'utiliser un masque complet avec cartouches à poussière quand on est exposé à de fortes concentrations de poussières lorsqu'une élimination à la source est impossible. Compte tenu de la non-disponibilité des masques adaptés aux poussières à cause de leur coût élevé, il est impératif d'avoir des locaux non fermés. Ceci pourrait être une solution temporaire pour nos meuniers artisanaux.

Cependant, le type d’étude transversale ne peut pas démontrer le lien causal entre l'exposition aux poussières des céréales, les manifestations respiratoires et l'environnement des meuniers artisanaux. D'où l'importance des études longitudinales pour bien investiguer la santé respiratoire des meuniers artisanaux congolais.

## Conclusion

Cette étude a détaillé l'impact négatif des poussières organiques provenant des céréales (manioc, maïs et soja) sur la santé respiratoire des meuniers à Lubumbashi. En plus de la nécessité de réglementer la profession, il est important que les décideurs en matière de politique sanitaire organisent un service de santé et de sécurité au travail qui puisse mettre en œuvre des mesures préventives destinées à réduire l'exposition aux poussières dans les meuneries artisanales et une surveillance médicale périodique des meuniers.

## Remerciements

Les auteurs remercient le Comité de gestion de l’Université de Kamina pour son appui administratif, le Comité des meuniers pour sa collaboration, l’équipe des enquêteurs (Dr Ivan Ngeleka, Dr Pierre Ntwadi, Dr Guy Banza) pour la réalisation de cette étude.

## Contribution des auteurs

Tous les auteurs ont contribué à la conception et à la rédaction de ce travail.

## Source de financement

Cette étude a bénéficié de l'appui matériel de la faculté de médecine de l’Université de Kamina, de l’Institut supérieur des sciences et techniques médicales de Lubumbashi et de l’Université de Kochi au Japon.

## Conflit d'intérêt

Les auteurs déclarent ne pas avoir de liens d'intérêts.
