# Supplementary material for: Exposure to cassava, corn and soybean dust and respiratory health among artisanal millers in Lubumbashi (Democratic Republic of the Congo)
Source: Med Trop Sante Int. 2024 Dec 11;4(4):mtsi.v4i4.2024.610. [Article in French] doi: 10.48327/mtsi.v4i4.2024.610 (PMC11892386; doi:10.48327/mtsi.v4i4.2024.610)
Supplement: Related texts

# Research article: Exposure to cassava, corn and soybean dust and respiratory health among artisanal millers in Lubumbashi (Democratic Republic of the Congo)

## Introduction

The respiratory effects of flour dust and other dust on individuals occupationally exposed in small and large milling industries have long been recognized [3, 35]. Respiratory diseases caused by environmental dust are influenced by the type of dust and the duration of exposure [22]. Several scientific reports suggest that lack of dust protection in agricultural environments can lead to pulmonary fibrosis [12, 32, 33].

Dust generated by grain handling may contain the following products: grain fragments (friction between grains, grinding debris, husks, etc.), pollen, bacteria (endotoxins), fungi (mycelium, spores, toxins), insects and invertebrates (grain insects, moths), mammalian and bird remains (hair, feathers, droppings), mineral dust (silica, etc.), pesticides [13]. Dust vary according to the type of grain, storage conditions, temperature and humidity.

On the other hand, the large amounts of dust found in the ambient air of artisanal mills are due, among other things, to outdated equipment, grain handling (especially during transport), grain spillage into machinery, the milling process, and flour bagging (Fig. 1, 2, 3 and 4). However, it is known that dust exposure in the working environment can be reduced or eliminated by improving processing equipment, ventilation systems, good working conditions and the use of personal protective equipment [10].

**Figure 1:** Outdated equipment in an artisanal mill (to be continued)

Comments: Presentation of outdated equipment and working environment of Congolese artisanal millers, which do not meet occupational health requirements. There's also a lot of grain dust everywhere.

**Figure 2:** Cassava milling machines

**Figure 3:** Front view of artisanal flour mill entrance

Comments: No ventilation windows, cereals are put into cut cans.

**Figure 4:** Soybean milling machines

Comments: Soybean milling machines. Outdated equipment, lots of soy dust in the unventilated room.

Exposure to organic dust and endotoxins causes various respiratory pathologies (asthma, alveolitis, chronic bronchitis, toxic pneumonitis) [11, 14, 19, 25] and acute or chronic respiratory failure [31, 34, 37, 38].

Cassava and maize flour are staple foods in the Democratic Republic of Congo (DRC) and other countries in the Central African region. As a result, milling has become a widespread and lucrative economic activity in the DRC. However, it is unregulated and poses a number of health risks. Milling typically takes place in enclosed, unventilated spaces with no means of protection from dust, mill vibrations, or combustion engine exhaust.

Respiratory symptoms and disorders among millers have been reported in the literature, but to our knowledge, no scientific study has been conducted or published on the respiratory status of millers in the DRC or Central Africa. The objective of this work was to determine the prevalence of respiratory symptoms and their determinants among artisanal millers exposed to cassava, maize and soybean dust in the city of Lubumbashi, Katanga Province, DRC.

## Materials and methods

### Study type, setting and sample

A cross-sectional study with analytical objectives was conducted in 2015 in the seven communes of the city of Lubumbashi, Katanga Province, over a period of three months, from May 1 to July 31, 2015.

The study population consisted of artisanal millers (exposed group) and agents of a local security company (control group) working in the same neighborhoods as the artisanal millers in the city of Lubumbashi [28]. In the absence of an occupational health service, respondents were recruited at their respective workplaces. A total of 510 artisanal mills were identified in the 7 communes of the city of Lubumbashi (each commune was considered a site) thanks to the support of neighborhood chiefs and miller representatives. Only 390 artisanal mills were operational during the study period. The sample size was based on the number of millers found at the site on each odd day of the week, counted from lists prepared by the neighborhood chiefs or representatives of the artisanal millers. We took an exhaustive sample of all millers in the city of Lubumbashi. A total of 350 artisanal millers with more than 6 months of experience in the trade were recruited for the study (including 2 women); of these, 288 who met the inclusion criteria (presence in the workplace on the day of recruitment, informed consent, and voluntary participation) were selected to participate in the survey. Each miller received an anonymous survey form, which was also used to record clinical examination data.

The control group consisted of 125 carefully recruited security guards with more than six months of service and a socioeconomic level similar to that of the artisanal millers. Seven controls did not agree to participate in the study and were excluded.

### Survey Questionnaire

The study included an analysis of working conditions (questionnaire, physical examination and spirometry). The workers were examined at their respective workplaces. Information on the health of the millers and controls was collected by means of a standardized questionnaire consisting of 7 items, including: a general questionnaire on sociodemographic and occupational characteristics, a questionnaire on respiratory symptoms [5], a questionnaire on allergic rhinitis [2], a standardized questionnaire on sleep and vigilance disorders related to the Epworth Sleepiness Scale [15], the Berlin questionnaire [27], a neurological questionnaire [21]. Only the questionnaire on respiratory symptoms was used in our study [2, 5].

### Definitions of target pathologies and functional airway examination

Chronic bronchitis was defined according to World Health Organization (WHO) criteria as chronic cough and sputum production for at least three months per year for at least two years. Rhinitis was classically characterized using a quantitative Score For Allergic Rhinitis (SFAR). Weight was measured with a calibrated and verified scale; height was measured with a tape measure. Blood pressure was recorded with an electronic device (OMRON Hem8402) placed on the right arm supported at heart level, with the subject resting in a seated position for at least 10 minutes.

Spirometry was performed on participants who freely consented to the test, including 160 millers and 90 controls. It was carried out outside the workshop using a portable “Easy One” device, which does not require calibration. Each subject made at least three consecutive attempts, and the best result was selected, taking into account the requirements of the ATS/ERS 2005 protocol. The following parameters were measured: forced vital capacity (FVC), forced expiratory volume in one second (FEV1), Tiffeneau ratio (FEV1/FVC), peak expiratory flow (PEF), median expiratory flow (MEF), maximum expiratory flow at 25, 50 and 75% of vital capacity (MEF25, MEF50, MEF75).

### Ethical considerations

Respondents were assured of confidentiality and anonymity. Interviewers were trained in advance and the survey was conducted by face-to-face interview between the interviewers and the subjects. Informed consent was obtained from subjects.

### Statistical Analysis of Study Data

EPI info7 software was used for data coding. All analyses were performed with Stata software (Stata Corporation Inc., Texas, USA). The statistical study was based on Student's T-test for comparison of means with respect to respiratory function parameters (spirometry) and Fisher's exact test for proportions with respect to anthropometric and sociodemographic characteristics. For categorical variables, such as respiratory symptoms, a cross-tabulation was performed, and chi-squared and relative risk values were estimated.

On the other hand, multivariate analysis using the logistic regression test was preferred to determine the association between miller characteristics and reported respiratory symptoms. This model estimates the strength of the association using a prevalence ratio (odds ratio, OR) and its 95% confidence interval (I.C.95%). The significance level chosen was p<0.05.

## Results

### Brief description of millers’ working conditions

In flour mills, millers are exposed to several types of exposures that may affect their health: dust from cassava flour and grains (maize, soy), vibrations and noise from mill motors, which are (mostly) electric and outdated, as well as heat and poor ventilation conditions due to the lack or insufficient number of windows. Millers work in different shifts (maize-cassava-soybean, maize-casava, maize, cassava-soybean, cassava alone) and generally in almost closed environments (without adequate ventilation). In some flour mills that use an internal combustion engine as a power source, exhaust gases are vented to the outside. It is important to note that all mills are fixed in enclosed spaces and cannot be moved under canopies. These artisanal mills have never respected the three stages of grain processing: cleaning, grinding and milling. This survey shows that 100% of the artisanal millers have no personal protective equipment at work.

The size of the study sample was 406 participants, including 288 millers and 118 controls, whose characteristics are presented in Table I.

**Table I:** Characteristics of participants

Characteristics of participants	Millers (N=288)	Controls (N=188)
Anthropometric, clinical and work-related parameters		
	Average +/- Standard deviation	Average +/- Standard deviation
age	27,6±9	28,5±7
seniority (years)	4,8±5,5	4,9±4,4#
working hours (hours)	12,1±1,7	14,4±6,2#
BMI	22,1±2,4	23,3±3,6#
SBP	140,3±15,9#	126,1± 17,4
DBP	90±12,1#	73,9±12,2
Socio-demographic parameters		
	n (%)	n (%)
civil status		
married	128 (44,4)	88 (74,6)*
single	158 (54,9)	28 (23,7)
divorced	2 (0,7)	0
widowed	0	2 (1,7)
education		
primary	3 (1,0)	5 (4,2)
secondary (incomplete)	244 (84,7)*	78 (66,1)
secondary (completed)	41 (14,2)	35 (29,7)
alcohol use		
yes	125 (43,4)*	17 (14,4)
no	163 (56,6)	101 (85,6)
smoking		
yes	90 (31,5)*	17 (14,8)
no	196 (68,5)	98 (85,2)

IMC : Indice de masse corporelle; PAS : Pression artérielle systolique; PAD : pression artérielle diastolique)

Notes : #, valeur de P inférieure à 0,01 pour le test « T » de Student; *, valeur de P inférieur à 0,001 pour le test exact de Fisher.

BMI: Body Mass Index; SBP: Systolic Blood Pressure; DBP: Diastolic Blood Pressure)

Notes: #, P value less than 0.01 for Student's ‘T’ test; *, P value less than 0.001 for Fisher's exact test

Table II shows the prevalence of respiratory symptoms in the millers and the control group.

**Table II:** Prevalence of respiratory symptoms, with corresponding risk, according to occupation

Respiratory symptoms	Millers (N=288)	Controls (N=188)	RR IC [95 %]	x^2^	P
Wheezing	124 (43,4)	27 (23,5)	1,88 [1,31-2,68]	14,58	< 0,001
Respiratory discomfort	100 (34,7)	20 (16,9)	2,05 [1,33-3,14]	12,70	< 0,001
Shortness of breath at rest	88 (30,6)	6 (5,1)	6,01 [2,70- 13,35]	30,52	< 0,0001
Shortness of breath on exertion	94 (32,6)	6 (5,1)	6,41 [2,89-14,24]	34,23	< 0,0001
Cough	121 (42,0)	23 (19,5)	5,25 [2,35-11,73]	24,13	< 0,0001
Morning sputum	99 (34,4)	8 (6,8)	5,07 [2,54-10,08]	32,84	< 0,0001
Chronic bronchitis	76 (26,4)	5 (4,2)	6,22 [2,58-15,00]	25,72	< 0,0001
Asthma	136(47,2)	3(2,5)	18,5 [6,03-57,14]	74,22	< 0,0001
Rhinitis	87 (30,2)	12 (10,2	3,82 [2,01-7,23]	18,23	< 0,0001

RR : Risque relatif; IC : Intervalle de confiance

RR: Relative risk; CI: Confidence interval

Table III shows the prevalence of each respiratory symptom, as reported by the millers, as a function of workplace and grain to which they are exposed. Table IV shows the association between miller occupation and respiratory symptoms after adjustment for factors affecting respiratory function: smoking, exposure duration (length of time in the occupation), age, education level, and body mass index.

**Table III:** Distribution of respiratory symptoms in traditional millers according to workstation

Respiratory symptoms	Maize-cassava-soybean (n=45) (%)	Maize-cassava (n=181) (%)	Maize (n=44) (%)	Cassava-soybean (n=2) (%)	Cassava (n=16) (%)	P
Wheezing						
yes	25 (55,6)	72 (39,8)	17 (38,6)	1 (50)	9 (56,3)	<0,01
no	20 (44,4)	109 (60,2)	27 (61,4)	1 (50)	7 (43,8)	
Respiratory discomfort						
yes	22 (48,9)	53 (29,3)	15 (34,1)	1 (50)	9 (56,3)	<0,01
no	23 (51,1)	128 (70,7)	29 (65,9)	1 (50)	7 (43,8)	
Shortness of breath at rest						
yes	10 (22,2)	56 (30,9)	16 (36,4)	1 (50)	5 (31,3)	<0,01
no	35 (77,8)	125 (69,1)	28 (63,6)	1 (50)	11 (68,8)	
Shortness of breath on exertion						
yes	17 (37,8)	58 (32)	12 (27,3)	0 (0)	7 (43,8)	<0,01
no	28 (62,2)	123 (68)	32 (72,7)	2 (100)	9 (56,3)	
Cough						
yes	22 (48,9)	78 (43,1)	16 (36,4)	0 (0)	5 (31,3)	<0,01
no	23 (51,1)	103 (56,9)	28 (63,6)	2 (100)	11 (68,8)	
Morning sputum						
yes	14 (31,1)	63 (34,8)	17 (38,6)	1 (50)	4 (25)	<0,01
no	31 (68,9)	118 (65,2)	27 (61,4)	1 (50)	12 (75)	
Sputum >3 months						
yes	8 (17,8)	47 (26)	13 (29,5)	0 (0)	8 (50)	<0,01
no	37 (82,2)	134 (74)	31 (70,5)	2 (100)	8 (50)	
Asthma						
yes	19 (42,2)	87 (48,1)	23 (52,3)	0 (0)	7 (43,8)	<0,01
no	26 (57,8)	94 (51,9)	21 (47,7)	2 (100)	9 (56,3)	

**Table IV:** Prevalence of respiratory symptoms, with corresponding risk, according to occupation (to be continued)

Variables	Wheezing		Respiratory discomfort		Shortness of breath at rest		Shortness of breath on exertion		Cough
	OR[IC95%]	P*	OR[IC95%]	P*	OR[IC95%]	P*	OR[IC95%]	P*	OR[IC95%]	P*
Age										
≤ 30	0,99[0,94-1,03]	0,70	0,99[0,95-1,04]	0,95	1,01[0,94-1,05]	0,98	2,75[1,23-6,14]	0,013	1,02[0,97-1,08]	0,37
> 30	1		1		1		1		1	
Seniority (years)										
≤ 5	1,25[0,76-2,03]	0,369	1,13[0,67-1,9]	0,64	1,07[0,59-1,94]	0,81	1,41[1,56-1,84]	0,043	0,59[0,30-1,14]	0,12
> 5	1		1		1		1		1	
Working hours (hours)										
≤ 12	0,93[0,85-1,03]	0,27	0,85[0,85-1,06]	0,43	1,06[0,84-1,17]	0,17	1,22[1,44-3,32]	0,69	1,66[0,74-4,43]	0,31
> 12	1		1		1		1		1	
Smoking										
yes	0,96[0,59-1,55]	0,87	1,23[0,73-2,05]	0,43	0,83[0,48-1,42]	0,51	1,30[1,22-2,27]	0,35	1,03[0,58-1,82]	0,90
no	1		1		1		1		1	
Occupation										
miller	2,47[1,48-4,11]	<0,01	2,75[1,56-4,83]	<0,01	7,85[3,27-18,84]	<0,01	3,69[9,57-24,81]	<0,01	7,16[2,96-17,29]	<0,01
control	1		1		1		1		1	
Education										
primary, secondary (incomplete)	0,87[0,52-1,46]	0,12	1,12[0,65-1,91]	0,67	0,71[0,37-1,38]	0,32	0,61[0,31-1,18]	0,14	1,13 [0,59-2,15]	0,7
secondary completed, more	1		1		1		1		1	

Variables	Morning sputum		Chronic bronchitis		Asthma		Rhinitis	
	OR[IC95%]	P*	OR[IC95%]	P*	OR[IC95%]	P*	OR[IC95%]	P*
Age								
≤ 30	1,02[0,97-1,08]	0,37	1,38[0,61-3,18]	0,40	1,03 [0,65-1,05]	0,88	0,88[0,46-1,71]	0,08
> 30	1		1		1		1	
Seniority (years)								
≤ 5	1,44[1,23-1,83]	<0,01	0,66[0,34-1,28]	0,224	1,07[0,60-1,89]	0,81	1,24[0,81-1,88]	0,83
> 5	1		1		1		1	
Working hours (hours)								
≤ 12	1,06[0,95-1,19]	0,27	1,10[0,96-1,25]	0,14	1,74[1,10-2,29]	<0,01	1,35[0,83-2,21]	0,49
> 12	1		1		1		1	
Smoking								
yes	1,38[1,80-2,38]	0,245	1,55 [1,85-2,82]	0,15	1,11[0,66-1,85]	0,69	0,99[0,67-1,47]	0,23
no	1		1		1		1	
Occupation								
miller	7,35[3,37-16,3]	<0,01	8,21[3,16-21,2]	<0,01	36,28[11,1-118,6]	<0,01	2,97[1,69-5,22]	0,001
control	1		1		1		1	
Education								
primary, secondary (incomplete)	0,54[0,28-1,05]	0,07	1,38[1,17-1,91]	0,017	1,27[0,69-2,33]	0,44	0,61[0,31-1,18]	0,144
secondary completed, more	1	1	1	1

Table V shows the trend in the values of the various respiratory function parameters in the millers and in the control group. None of the millers participating in the study wore personal protective equipment at work.

**Table V:** Comparison of spirometric parameters between millers and controls

Spirometrie parameters	Millers (Mean±SD)	Control (Mean±SD)	T	P (T-test)
FVC (L)	4,02±0,72	4,25±0,14	0,36	0,359
FEV1 (L)	3,29±0,59	3,35±0,71	2,71	0,039
FEV1/FVC (% predicted)	82,3 5±8,31	85,81±8,12	4,95	< 0,001
PEF (L/s)	8,12±1,97	8,63±1,62	1,31	0,095
PEF (% predicted)	93,24±21,91	101,82±17,96	2,01	0,023

CVF : Capacité vitale forcée; VEMS: Volume expiratoire maximal seconde; DEP : Débit expiratoire de pointe

FVC : Forced vital capacity; FEV1: Forced expiratory volume; in 1 second PEF : Peak expiratory flow

## Discussion

This study showed that the prevalence of respiratory symptoms and pathologies was remarkably high among artisanal millers exposed to cassava, maize and soybean dust compared to the control group in the city of Lubumbashi. It has been shown that workers exposed to dust may also inhale spores or fragments of mycelium [8, 30], which increases the risk of developing respiratory symptoms [4, 6, 7, 20, 36]. The study showed that artisanal millers did not have the necessary protective equipment (goggles, masks, gloves), often due to lack of funds, training or information, or the unavailability of the latter. All these factors contribute to the high prevalence of respiratory symptoms and pathologies among Congolese artisanal millers. Our observations are supported by the literature [11, 22, 25], which highlights the lack of education, low socio-economic level and unavailability of protective equipment in the informal sector, particularly among quarry workers, including those in the agricultural sector.

Furthermore, our results are comparable to those of previous studies conducted in the DRC among different groups of dust-exposed workers [1, 10, 26, 29]. However, the prevalence of asthma in our study was much higher than that reported by some authors [9, 24], suggesting that asthma is a common disease among workers exposed to cereal dust.

An association was found between the occupation of artisanal millers and their respiratory symptoms. According to the literature, volatile organic compounds (VOCs) play a role in the deterioration of the respiratory tract, while high concentrations of fine particles are responsible for alterations in the state of health of subjects exposed to environmental dust [2, 31, 34].

The unhealthy environment (with the presence of large amounts of dust in the premises and even on the machines), poor and unhygienic conditions (cramped premises, lack of ventilation, congestion, etc.), exposure to grain dust could also explain this association [29]. This risk could be increased by exhaust fumes if they are not properly vented to the outside, which we could not take into account in our analyses. Similarly, smoking may influence the incidence, severity, and spontaneous evolution of many respiratory diseases [9, 16, 17, 24]. In our study, a high prevalence of tobacco and alcohol use was found among artisanal millers compared to the control group. This phenomenon is common among bonded laborers working under precarious conditions. This observation has also been made by some authors [29]. The logistic regression test suggests a negative impact of tobacco and dust exposure on the respiratory health of artisanal millers.

The significant reduction in respiratory function, particularly in parameters such as FEV1 and PEF, in the artisanal millers compared with the control group would reflect the hypertrophy of mucous cells due to dust irritation, resulting in increased mucus secretion, mucus plug formation and obstruction of exhaled air [18, 23, 29]]. Organic dusts vary according to the nature and chemical composition of each product, which may influence the incidence of respiratory symptoms in exposed workers. Our study suggests that exposure to cassava and maize dusts is more toxic to the respiratory system than soybean dusts. Further studies are required to determine the mechanism.

This work is important because it provides health policy makers and researchers with relevant information on the prevalence of respiratory manifestations, the work environment, and the lack of preventive measures among Congolese artisanal millers.

It is important to reduce the concentration of grain dust in the millers’ working environment, to integrate ventilation and dust extraction systems in the workshops, to improve hygiene measures, and to define our dust exposure limits and the dimensions of artisanal milling workshops in the DRC.

It is also essential to use a full-face mask with dust cartridges when exposed to high concentrations of dust and when elimination at source is not possible. Given the unavailability of dust masks due to their high cost, it is imperative to have unenclosed spaces. This could be a temporary solution for our artisanal millers.

However, cross-sectional studies cannot establish a causal relationship between grain dust exposure, respiratory symptoms and the environment of artisanal millers. Therefore, longitudinal studies are important to properly investigate the respiratory health of Congolese artisanal millers.

## Conclusion

This study has detailed the adverse effects of organic grain dust (cassava, maize and soybean) on the respiratory health of millers in Lubumbashi. In addition to the need to regulate the profession, it is important for health policy makers to organize an occupational health and safety service that can implement preventive measures to reduce dust exposure in artisanal mills and provide regular medical surveillance of millers.

## Acknowledgments

The authors would like to thank the Management Committee of the University of Kamina for its administrative support, the Millers’ Committee for its cooperation, and the research team (Dr. Ivan Ngeleka, Dr. Pierre Ntwadi, Dr. Guy Banza) for carrying out this study.

## Authors’ contributions

All the authors contributed to the design and writing of this work.

## Funding

This study received material support from the Faculty of Medicine, University of Kamina, the Institut supérieur des sciences et techniques médicales de Lubumbashi and the University of Kochi, Japan.

## Conflict of interest

The authors declare no conflict of interests.
